# Immune response to allergens in sheep sensitized to house dust mite

**DOI:** 10.1186/1476-9255-5-16

**Published:** 2008-10-20

**Authors:** Robert J Bischof, Ken J Snibson, Joanne Van Der Velden, Els NT Meeusen

**Affiliations:** 1 Animal Biotechnology Research Laboratories, Department of Physiology, School of Biomedical Sciences, Monash University, Clayton, 3800, Australia; 2 Centre for Animal Biotechnology, School of Veterinary Science, The University of Melbourne, Victoria, 3010, Australia

## Abstract

**Background:**

House dust mite (HDM) allergens are a major cause of allergic asthma. Most studies using animal models of allergic asthma have used rodents sensitized with the 'un-natural' allergen ovalbumin. It has only recently been recognized that the use of animal models based on HDM provide a more relevant insight into the allergen-induced mechanisms that underpin human allergic disease. We have previously described a sheep model of human allergic asthma that uses *Dermatophagoides pteronyssinus *HDM. The present study extends our understanding of the immune effects of HDM and the allergens Der p 1 and Der p 2 in the sheep model of asthma.

**Methods:**

Peripheral blood sera from non-sensitized (control) sheep and sheep sensitized to HDM was collected to determine immunoglobulin (Ig) reactivities to HDM, Der p 1 and Der p 2 by ELISA. Bronchoalveolar lavage (BAL) fluid collected following allergen challenge was also assessed for the presence of HDM-specific antibodies. To examine the cellular immune response to HDM allergens, T cell proliferation and cutaneous responses were assessed in sensitized and control sheep.

**Results:**

Strong HDM- and Der p 1-specific IgE, IgG_1_, IgG_2 _and IgA serum responses were observed in sensitized sheep, while detectable levels of HDM-specific IgG_1 _and IgA were seen in BAL fluid of allergen-challenged lungs. In contrast, minimal antibody reactivity was observed to Der p 2. Marked T cell proliferation and late phase cutaneous responses, accompanied by the recruitment of eosinophils, indicates the induction of a cellular and delayed-type hypersensitivity (DTH) type II response by HDM and Der p 1 allergen, but not Der p 2.

**Conclusion:**

This work characterizes the humoral and cellular immune effects of HDM extract and its major constituent allergens in sheep sensitized to HDM. The effects of allergen in HDM-sensitized sheep were detectable both locally and systemically, and probably mediated via enzymatic and immune actions of the major HDM allergen Der p 1. This study extends our understanding of the actions of this important allergen relevant to human allergic asthma and its effects in sheep experimentally sensitized to HDM allergens.

## Background

Many proteins of the house dust mite (HDM) *Dermatophagoides pteronyssinus *are potent enzymes and represent the most important allergens associated with human allergic asthma [1]. The most extensively studied HDM allergens are Der p 1 and Der p 2 and it has been shown that the majority of HDM-sensitized asthmatic patients (80â€“100%) have strong serum IgE responses to these allergens [2].

The immunological and direct biological effects of HDM allergens have been well documented in recent years. Local and systemic immune effects of HDM allergens include recruitment and activation of immune cells, release of inflammatory mediators and the up-regulation of pro-inflammatory adhesion molecules [3-5]. Der p 1, the most immunodominant and widely studied HDM allergen, is a cysteine protease with reported immune and enzymatic effects in allergic human asthma [1]. Der p 1 proteolytic activity is thought to be a major contributor to its allergenicity. Some of the reported actions of Der p 1 include direct immunomodulatory effects through cleavage/down-regulation of CD23 on B cells [6], CD25 on T cells [7] and CD40 on dendritic cells [8], as well as the disruption of tight junctions in the bronchial epithelium leading to increased cell permeability [9].

Most studies using animal models of allergic asthma have used rodents and are based on sensitization and challenge with the 'un-natural' allergen ovalbumin. With mounting evidence for the potent role of HDM allergens in shaping immune responses in the tissue microenvironment, there is a need for more animal models that utilize the HDM allergens as a more relevant model for the human disease [1,10]. The development of *in vivo *animal models of experimental asthma based on HDM allergens has raised further interest in exploring the specific roles that natural allergens play in allergic disease. HDM effects have been investigated in small animal models of asthma [11-16], while previous studies in our laboratory have reported the effects of HDM in a sheep model of allergic asthma [17-20].

The HDM sheep asthma model displays many of the characteristic features of human allergic asthma including HDM-specific IgE responses, eosinophilia, mucus hypersecretion of the airways, and airway remodeling following chronic allergen exposure. A proportion of HDM allergic sheep also develop increased airway resistance and airway hyperreactivity similar to human asthmatics [20], validating the suitability of this experimental sheep asthma model. The present study was undertaken to extend our knowledge of the cellular and immune responses induced by HDM and its major allergens, Der p 1 and Der p 2, in sheep sensitized to HDM.

## Methods

### House dust mite (HDM) allergens

Whole extract of the *Dermatophagoides pteronyssinus *house dust mite (HDM), was obtained from CSL Limited (VIC, Australia), prepared in pyrogen-free saline (PFS; Baxter Healthcare Pty. Ltd, NSW, Australia) and stored at -70Â°C prior to use [17]. The concentration of HDM extract used in the studies detailed below was based on the amount of the crude HDM extract prepared in PFS. Immuno-affinity purified Der p 1 obtained from cultured mites was kindly provided by Dr Alan Brown (University of Nottingham, UK). Recombinant Der p 2 was a generous gift from Dr Wayne Thomas (University of Western Australia, Australia).

### Experimental sheep and sensitization with HDM

All experimental animal procedures and the collection of tissues and cells were approved by the Animal Experimentation Ethics Committee of the University of Melbourne, following guidelines set by the National Health and Medical Research Council (NH&MRC) of Australia. Female Merino-cross lambs (4â€“5 months of age) used in these studies were treated with the anthelminthic Nilvermâ„¢ (Cooper's Animal Health, NSW, Australia) to eliminate any possible parasite infections prior to the experiment and were housed in pens and fed *ad libitum*. All sheep were judged free of significant pulmonary disease on the basis of clinical examination.

Sheep (n = 8) were sensitized to HDM following 3 immunizations with solubilized HDM (50 Î¼g in aluminium hydroxide) as outlined previously [17]; control (naÃ¯ve) animals (n = 6) were not immunized with HDM. Peripheral blood samples were collected from controls and at pre- and post-immunization and stored frozen prior to determination of immunoglobulin (Ig) reactivity. Immunized animals that showed increased HDM-specific serum IgE levels as assessed by ELISA (6 of the 8 immunized sheep) were classified as allergic [17] and used as sensitized sheep in the experiments.

### Airway allergen challenges and collection of bronchoalveolar lavage (BAL) samples

Four HDM-sensitized sheep were rested for 3 weeks following the final immunization, then given 2 airway challenges with HDM allergen (whole extract) at 3 week intervals, using a fibre-optic bronchoscope as outlined previously [17]. Bronchoalveolar lavage (BAL) samples were collected 1 week before (0 h baseline timepoint) and 48 h after instillation of HDM allergen into the airways using a fibre-optic bronchoscope [17], cells separated by centrifugation, and BAL supernatants frozen for later determination of antibody reactivity.

### Detection of antibody responses to HDM allergens

Conventional enzyme-linked immunosorbent assay (ELISA) was used for the detection of IgE, IgG_1 _and IgG_2_, IgA and total Ig against HDM allergens in peripheral blood serum and BAL samples, as detailed previously [17]. Briefly, ELISA plates coated with HDM (0.5â€“10 Î¼g/well), Der p 1 (0.75 Î¼g) or Der p 2 (0.75 Î¼g), were blocked and washed prior to incubation with triplicate serum samples (diluted 1/1000). For the IgE ELISA, serum samples were NH_4_SO_4_-treated prior to analysis as described [17]. Following washes, plates were incubated with anti-ovine IgG_1 _or IgG_2 _(clone 7732, 4243 [21]), anti-ovine IgE (YD3/XB6 [22]) or anti-bovine IgA (VMRD Inc., WA, USA) mAb, then washed and incubated with horseradish peroxidase (HRP)-conjugated rabbit anti-mouse Ig (Dako, CA, USA). For total Ig determination, HDM-coated plates were incubated with HRP-conjugated swine anti-sheep Ig (Dako). Plates were then washed, developed with 3', 3', 5', 5'-tetramethyl-benzidine dihydrochloride hydrate (TMB; Sigma, NSW, Australia) and optical density (OD) determined with a TitreTek Multiscan MCC plate reader using a dual wavelength (A_450_-A_690_).

### Proliferation assays

Peripheral blood was drawn from the jugular vein of sensitized and control animals and placed into a collecting tube containing ethylamine tetra-acetic acid (EDTA). Red blood cells were lysed with the addition of Tris-buffered ammonium chloride (TAC; 0.17 Î¼M Tris, 0.16 Î¼M NH_4_Cl, pH 7.2) at 39Â°C. White blood cells were layered over Ficoll (Sigma), peripheral blood mononuclear cells (PBMCs) collected from the interface and washed twice with sterile phosphate-buffered saline (PBS), then resuspended in DMEM tissue culture media (Invitrogen, Life Technologies, VIC, Australia) supplemented with 10% foetal bovine serum (FBS), penicillin, streptomycin, L-glutamine and 2-mercaptoethanol (4 Î¼M).

PBMCs collected from sensitized and control animals were plated out into 96-well flat-bottom plates at 5 Ã— 10^5 ^cells/well in a volume of 200 Î¼l. Antigens were added in triplicate at 10 Î¼g/ml and cells were incubated at 37Â°C/5% CO_2 _for 4 days. Control cultures containing no antigen or concanavalin A (conA, 1 Î¼g/ml; Sigma) were also included. DNA synthesis was measured with the addition of ^3^H thymidine (0.5 Î¼Ci; DuPont, Wilmington, DE, USA) during the final 16â€“18 h of culture. Cultures were harvested onto glassfibre filter mats and thymidine incorporation measured using a Packard beta counter. Results are presented as counts per minute (c.p.m.).

### Intradermal skin testing and dermal biopsy samples

Immediate and delayed cutaneous responses were assessed in sensitized and control sheep following intradermal (i.d.) injection with 100 Î¼l of either HDM (100 Î¼g/ml of crude extract), Der p 1 (5 Î¼g/ml) or Der p 2 (5 Î¼g/ml) in PBS. Control sites included challenge with PBS, ovalbumin (10 Î¼g/ml; Sigma) in PBS as irrelevant antigen and histamine diphosphate (10 Î¼g/ml; Sigma) as a positive control. Sheep were restrained while duplicate intradermal injections were made on the clipped flank using a 29 *g *needle/BD Ultra-Fineâ„¢ insulin syringe (BD, NJ, USA). Wheal reactions were scored at 0.25, 0.5, 1, 3, 5, 24, and 48 h post-challenge, calculated as the mean of two measurements of the wheal diameters made at right angles to each other using calipers.

Dermal (skin) biopsy samples were collected from an un-injected site as a control, and from one of each of the injection sites at 6 h and again at 48 h, fixed in 4% paraformaldehyde (PFA) in PBS and processed to paraffin for histology and immunochemistry.

### Histology and immunochemistry of dermal tissues

Paraffin-processed dermal tissue samples collected from sensitized and control animals were serial-sectioned (5 Î¼m) for histological staining and immunochemistry.

Eosinophil counts were determined in paraffin sections stained with Giemsa stain (Sigma). Mast cells were detected in paraffin sections by immunochemical staining [23] using a polyclonal rat anti-ovine tryptase antibody, kindly provided by Prof Hugh Miller (University of Edinburgh, UK); all sheep dermal and lung mast cells are tryptase-positive [24]. Briefly, PFA-fixed tissue sections were incubated with 10% FBS in PBS to block non-specific binding sites, followed by incubation with anti-ovine tryptase Ab (1:250 in PBS), then washed and incubated with HRP-conjugated rabbit anti-rat Ig (Dako). Sections were developed with 3,3'-diaminobenzidine tetrahydrochloride (DAB; Sigma) and counterstained with H&E.

Eosinophils and mast cells were counted in a minimum of 20 successive graticule squares, an area of 1.25 mm^2^, using a light microscope at 400Ã— magnification. Cells were counted in the upper dermis and lower dermis (area lying between the skin glands and deeper muscle layer), and intravascular cells were excluded from the count.

### Statistical analyses

Parametric statistical analyses of the data was carried out using Student's *t*-test to determine any significant differences (*p *< 0.05) between means of sample groups.

## Results

### Antibody responses to HDM and the dust mite allergens Der p 1 and Der p 2

**Figure 1:**
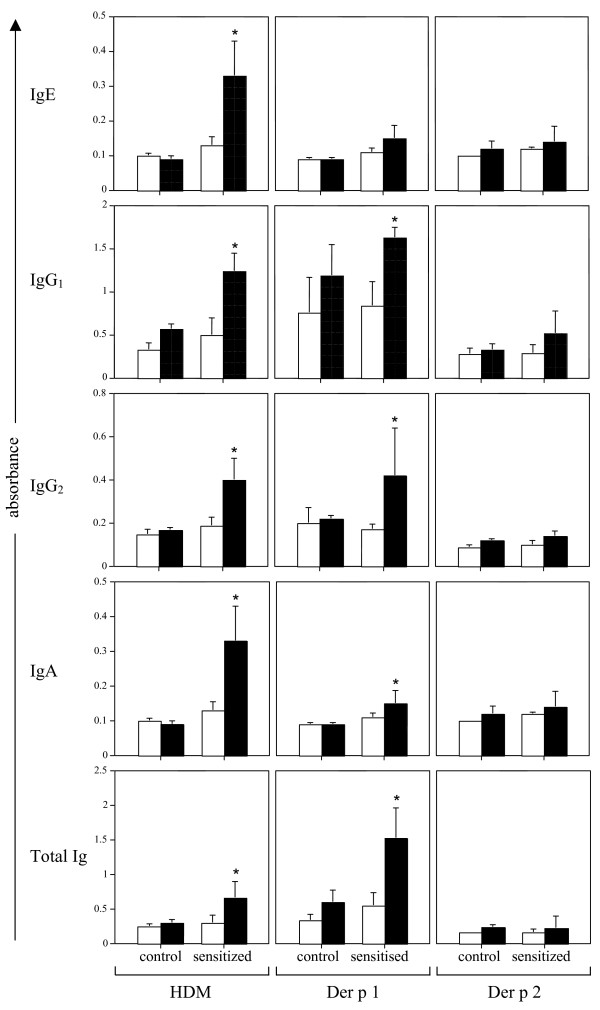
**Systemic IgE, IgG_1_, IgG_2_, IgA and total Ig antibody reactivities to HDM extract, Der p 1 and Der p 2 in serum from control and HDM-immunized sheep**. Peripheral blood serum samples were collected before (open bars) and 7 days after (closed bars) the final HDM injection, with time-matched serum samples also collected from control animals. In each of the plots, ELISA data is presented as mean absorbance values Â± s.d. * indicates significant differences (*p *< 0.05) comparing samples collected before and after HDM exposure; n = 3â€“4 per group.

ELISAs were performed to determine IgE, IgG_1_, IgG_2_, IgA and total Ig reactivities with HDM extract and the allergens Der p 1 and Der p 2 in serum samples from HDM-immunized and control (non-immunized) sheep. Sheep were classified as sensitized (allergic) on the basis of increased HDM-specific serum IgE levels. HDM-immunized animals displayed a greater than 2-fold increase in mean serum HDM-specific IgE levels compared to control sheep (Fig. 1). A significant increase in mean levels of HDM-specific IgG_1_, IgG_2_, IgA and total Ig was also detected in immunized compared to control sheep. Serum antibody reactivity to the HDM allergens Der p 1 and Der p 2 appeared more variable than that seen with the HDM preparation. Mean antibody levels were elevated (*p *< 0.05) for Der p 1-specific IgG_1_, IgG_2_, IgA and total Ig in immunized compared to controls, while no Der p 2 specific antibodies were detected in any of the serum samples assayed (Fig. 1).

**Figure 2:**
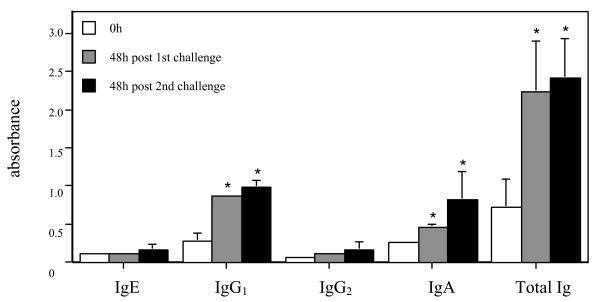
**Antibody release into BAL fluid following airway allergen challenge of HDM-sensitized, allergic sheep**. Antibody reactivity to HDM was assessed in BAL fluid collected prior to the first airway challenge (0 h) and 48 h following two separate airway challenges with HDM allergen (n = 4 allergic animals). ELISA data is presented as mean absorbance values Â± s.d. * indicates a significant difference (*p *< 0.05) compared to 0 h BAL samples.

BAL samples were collected before and after airway allergen challenges in HDM-sensitized sheep, and assayed by ELISA for HDM-specific IgE, IgG_1_, IgG_2_, IgA and total Ig (Fig. 2). The levels of IgG_1_, IgA and total Ig were all elevated (*p *< 0.05) at 48 h following the first, and similarly the second, airway challenge compared to baseline (0 h). In contrast, HDM-specific IgE and IgG_2 _in BAL fluid showed little change above baseline in the 4 animals tested.

### T cell proliferative responses to HDM and Der p 1 are elevated in sensitized sheep

**Figure 3:**
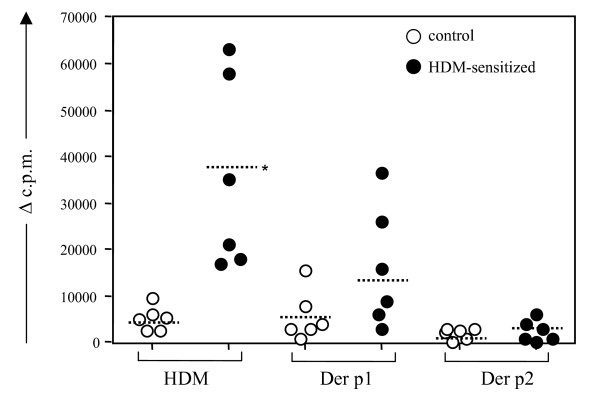
**HDM allergens induce the proliferation of blood lymphocytes from allergic sheep**. Proliferative response of PBMCs from control (open circles; n = 6) and HDM-sensitized (closed circles; n = 6) sheep following stimulation *in vitro *with HDM extract and the allergens Der p 1 and Der p 2. Data plotted as change in counts per minute from baseline (Î” c.p.m.). Dotted horizontal lines depict the mean value for each group; * indicates significance (*p *< 0.05) for the corresponding allergens in sensitized compared to control sheep.

PBMCs from HDM-sensitized and control sheep were stimulated with HDM or the HDM allergens Der p 1 and Der p 2 (Fig. 3). Strong T cell proliferative responses to HDM extract were observed in PBMCs from all sensitized sheep, in contrast to the control animals where the cell responses were minimal or negligible. A similar response pattern was seen following stimulation with Der p 1, although in the sensitized animals these responses were weaker compared to HDM. The Der p 2 allergen had no significant effect on T cell proliferation.

### Cutaneous hypersensitivity responses to HDM allergens

**Figure 4:**
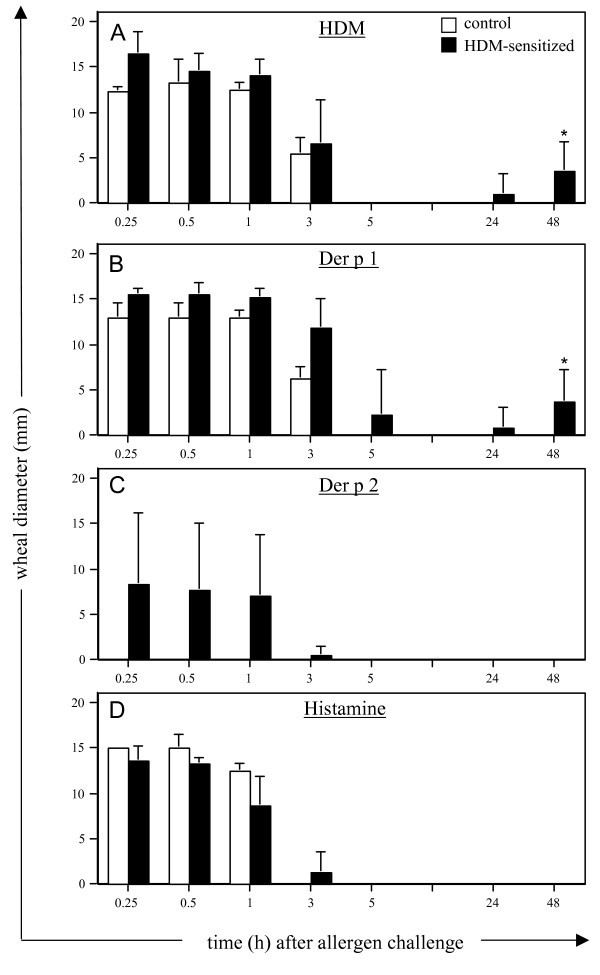
**Skin wheal reactions in response to HDM allergens**. Mean wheal reaction size measured at various time-points following intradermal challenge in control (n = 4) and HDM-sensitized (n = 5) sheep with (A) HDM extract, (B) Der p 1, (C) Der p 2 and (D) histamine. Values presented are means Â± s.d.; * indicates a significant difference (*p *< 0.05) comparing skin responses in sensitized versus control animals.

Skin reactions following cutaneous challenge with HDM, Der p 1 and Der p 2 were assessed in control and HDM-sensitized sheep. Immediate skin wheal reactions were observed within 15 minutes following injections with HDM extract and Der p 1 in all sheep (Fig. 4A and 4B). An immediate response to Der p 2 was elicited in 3 of the 5 sensitized sheep examined but absent in all controls (Fig. 4C). These acute responses, of similar magnitude to that elicited by histamine (Fig. 4D), persisted through the first hour post-challenge before declining over the 3â€“5 h period. Delayed-type hypersensitivity (DTH) responses were observed at 24â€“48 h for HDM extract and Der p 1 in sensitized but not in the control sheep (Fig. 4A and 4B). There was no indication of a DTH response following intradermal challenge with Der p 2 (Fig. 4C).

### Cellular changes in skin following challenge with HDM allergens

**Figure 5:**
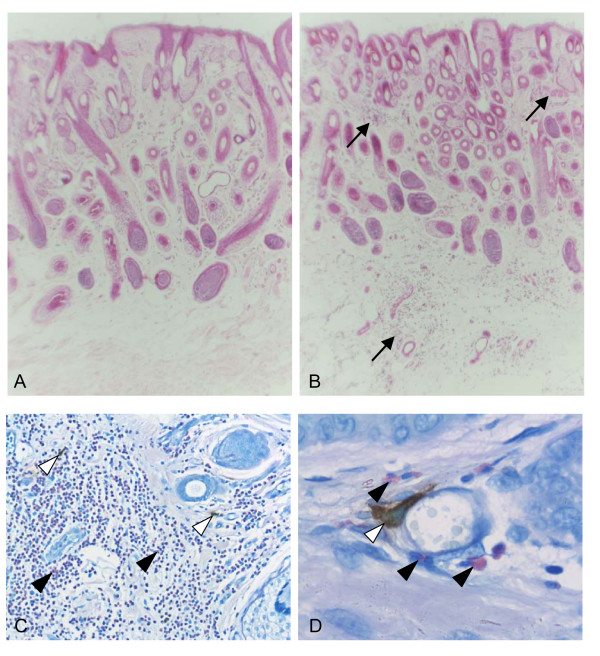
**Histology of skin biopsies following intradermal challenge with HDM allergens**. Histological staining of skin from (A) control and (B-D) HDM-sensitized sheep 48 h following cutaneous challenge with whole HDM extract shows extensive infiltration of eosinophils into the upper and lower dermal regions in skin of sensitized sheep (arrows); H&E stain (A, B), original magnification Ã—100. (C, D) Immunostaining for tryptase-positive mast cells in skin of sensitized sheep shows scattered mast cells (open arrowheads) together with eosinophils (arrowheads) throughout the dermis and in close proximity to blood vessels; Giemsa counterstain, original magnification Ã—200, Ã—1000).

Histological examination of tissue sections prepared from skin biopsies at 48 h following intradermal challenge with HDM allergens showed a prominent influx of eosinophils into the upper and lower dermis in skin of HDM-sensitized compared to control sheep (Fig. 5). Dermal tissue sections stained for mast cell tryptase showed positive staining of mast cells throughout the dermis, with mast cells distributed sporadically in the stroma and in close proximity to blood vessels (Fig. 5Câ€“D).

**Figure 6:**
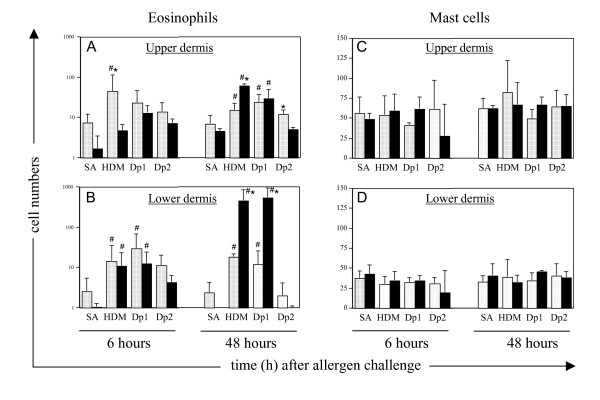
**Eosinophil and mast cell counts in skin of control and HDM-sensitized sheep following intradermal challenge with HDM allergens**. Eosinophils and tryptase-positive mast cells were counted in paraffin sections of skin collected from control (n = 4, open bars) and HDM sensitized (n = 5, closed bars) sheep at 6 h and 48 h following cutaneous challenge with saline (SA), house dust mite extract (HDM) or the allergens Der p 1 (Dp 1) and Der p 2 (Dp 2). Cells were counted in the upper and lower regions of the dermis and results expressed as the mean counts Â± s.d.; ^#^indicates a significant difference (*p *< 0.05) in cell counts compared to SA challenge; * indicates significance (*p *< 0.05) comparing sensitized and control tissues.

The enumeration of eosinophils and mast cells in sensitized and control tissues confirmed that there was a significant recruitment of eosinophils into the deeper dermal layers following allergen but not saline challenge (Fig. 6). This could already be detected at the 6 h time-point in the lower dermis of both sensitized and control sheep, but showed a further increase at 48 h post-allergen challenge in sensitized sheep only. This was coincident with the DTH responses described earlier. At 48 h post-challenge, tissue eosinophil numbers, in both sensitized and control tissues, were greater (*p *< 0.05) in both upper and lower regions of the dermis following HDM and Der p 1 compared to saline injection alone. In contrast to eosinophils, mast cell numbers did not vary with treatment or time-point in sensitized and control tissues.

## Discussion and conclusion

We have previously described a sheep model for human allergic asthma based on sensitization and challenge with *Dermatophagoides pteronyssinus *house dust mite (HDM) allergens [17,19,20]. In the present study we investigated the local and systemic immune effects of HDM and the major HDM allergens Der p 1 and Der p 2 in sheep.

The HDM allergens have been characterized and studied in detail *in vitro*, and we are only beginning to appreciate the need to focus on the direct and indirect mechanisms through which these relevant allergens direct the pathophysiology of allergic disease through *in vivo *models. The present study shows that sheep sensitized to HDM allergens display cellular and immune responses to HDM as well as to Der p 1, the major HDM allergen. The local and systemic effects of Der p 1 were generally found to parallel HDM with respect to the induction of HDM-specific antibodies, T cell proliferation and cellular recruitment following cutaneous challenge. This would suggest that in the sheep asthma model the effects of HDM may largely be mediated through Der p 1. In comparison to Der p 1, there was little response seen with Der p 2. This may have been due to non- or low reactivity to this allergen in sheep, or it may be that the recombinant Der p 2 used in the present study is not expressed in the appropriate conformation. In addition, previous studies have shown considerable polymorphism of Der p 2 allergens resulting in variable antibody and T cell responses [25].

The investigation of HDM-specific BAL Ig levels revealed a significant local Ig response, with a predominance of IgG_1 _and IgA, in allergen-challenged lungs. IgG_2 _and IgG_1 _antibody isotypes in ruminants are generally associated with a type-1 or type-2 immune response respectively (reviewed in [26]). The appearance of IgA antibodies is not surprising given that the role of IgA at mucosal surfaces and in respiratory disease has been well acknowledged [27,28]. The low levels of allergen-specific IgE in BAL fluid compared to serum samples is not unlike that seen in human subjects, where it has been reported that BAL specific IgE may not be detected in all allergic individuals, with lower levels seen in BAL fluid compared to serum [29]. The sheep asthma model may serve as a useful tool to further investigate the kinetics of local allergen-specific antibody release into the BAL fluid following allergen challenge and clarify the role of the different Ig subclasses through the early and late-phases of the asthmatic response.

Lymphocytes from sensitized sheep showed strong proliferative responses to HDM extract and to some extent Der p 1, suggesting the generation of Der p 1-specific memory T cells after HDM sensitization and their involvement in allergic responses in sheep. The comparatively weaker responses to Der p1 may be due in part to the amounts of allergen used (crude HDM extract versus purified Der p 1) in these proliferation studies. The effects of HDM allergens on T cell proliferation, activation and cytokine release, has been reported in animal models and human studies [30-33] and supports a Th_2_-driven mechanism in allergic asthma.

Skin reactions observed following injection of HDM extract and the allergens Der p 1 and Der p 2 in sensitized sheep suggest an immediate hypersensitivity response, generally attributed to degranulation of mast cells following the crosslinking of IgE with allergen. However, an immediate reaction following cutaneous HDM and Der p 1 challenge was also observed in control sheep, suggesting a non-specific stimulation by HDM allergens. This could be due to low levels of endotoxin known to be present in natural and commercial house dust mite preparations and shown to be a requirement for the development of allergic lung inflammation [34]. It is likely that, mast cell release of cytokines, inflammatory mediators and/or chemotactic factors triggered the rapid appearance of eosinophils to the site of inflammation within the first 6 h, as part of an acute, non-specific response to allergen challenge [35-37]. It is noteworthy that Der p 1 is capable of provoking cytokine release by mast cells in the absence of IgE [38]. No change was seen in the number of mast cells between HDM challenged and saline challenged skin of either control or sensitized sheep. Previous studies also did not observe a change in mast cell numbers after allergen provocation of allergic patients, and attributed this to the rapid replenishment of degranulated mast cells by migration of mast cells from the blood vessels [39]. In the sheep asthma model we have shown that mast cells are only significantly increased in the airways after chronic allergen exposure [19] and most likely play a significant role in the airway inflammation and remodeling changes. In the present study, the effects of a single cutaneous allergen administration were more discernible in sensitized sheep at 48 h post-challenge, with the appearance of a DTH reaction to HDM and Der p 1. The skin DTH observations were supported histologically with the recruitment of eosinophils into the dermis, a characteristic feature of a DTH type II response [40], and indicative of the more specific effects of allergen mediated via Th_2 _driven immune mechanisms at this later time point.

In this study we investigated the cell-mediated and humoral immune effects of HDM extract and its constituent allergens Der p 1 and Der p 2 in sensitized (allergic) compared to control, non-sensitized sheep. Both HDM and Der p 1 were shown to have systemic and local immune effects in sensitized sheep, and it is plausible to suggest that the majority of effects of HDM are mediated through its major allergen Der p 1. This study extends our understanding of the allergic status of sheep experimentally sensitized to house dust mite allergens, and the action of this important and relevant allergen in modulating an immune response.

## Competing interests

The authors declare that they have no competing interests.

## Authors' contributions

RJB conceived the study, carried out the majority of the experimental work and data analysis and drafted the manuscript. KJS assisted with some of the experimental procedures. JVV performed the tissue cell counts. ENTM participated in experimental design, data analysis and manuscript preparation. All authors read and approved the final manuscript.
